# Palmitic Acid Methyl Ester Induces G_2_/M Arrest in Human Bone Marrow-Derived Mesenchymal Stem Cells via the p53/p21 Pathway

**DOI:** 10.1155/2019/7606238

**Published:** 2019-12-01

**Authors:** Jian-Hong Lin, Pei-Ching Ting, Wen-Sen Lee, Hung-Wen Chiu, Chun-An Chien, Chin-Hung Liu, Li-Yi Sun, Kun-Ta Yang

**Affiliations:** ^1^PhD Program in Pharmacology and Toxicology, School of Medicine, Tzu Chi University, No. 701, Sec. 3, Zhongyang Rd., Hualien, Taiwan; ^2^Department of Surgery, Buddhist Tzu Chi General Hospital, No. 707, Sec. 3, Zhongyang Rd. Hualien, Taiwan; ^3^Graduate Institute of Medical Sciences, College of Medicine, Taipei Medical University, No. 250, Wuxing St., Taipei, Taiwan; ^4^Master Program in Medical Physiology, School of Medicine, Tzu Chi University, No. 701, Sec. 3, Zhongyang Rd., Hualien, Taiwan; ^5^Department of Molecular Biology and Human Genetics, Tzu Chi University, No. 701, Sec. 3, Zhongyang Rd., Hualien, Taiwan; ^6^Department of Pharmacology, School of Medicine, Tzu Chi University, No. 701, Sec. 3, Zhongyang Rd., Hualien, Taiwan; ^7^Gene and Stem Cell Manufacturing Center, Buddhist Tzu Chi General Hospital, No. 707, Sec. 3, Zhongyang Rd., Hualien, Taiwan; ^8^Department of Physiology, School of Medicine, Tzu Chi University, No. 701, Sec. 3, Zhongyang Rd., Hualien, Taiwan

## Abstract

Bone marrow-derived mesenchymal cells (BM-MSCs) are able to differentiate into adipocytes, which can secrete adipokines to affect BM-MSC proliferation and differentiation. Recent evidences indicated that adipocytes can secrete fatty acid metabolites, such as palmitic acid methyl ester (PAME), which is able to cause vasorelaxation and exerts anti-inflammatory effects. However, effects of PAME on BM-MSC proliferation remain unclear. The aim of this study was to investigate the effect of PAME on human BM-MSC (hBM-MSC) proliferation and its underlying molecular mechanisms. hBM-MSCs were treated with PAME for 48 h and then subjected to various analyses. The results from the present study show that PAME significantly reduced the levels of G_2_/M phase regulatory proteins, cyclin-dependent kinase 1 (Cdk1), and cyclin B1 and inhibited proliferation in hBM-MSCs. Moreover, the level of Mdm2 protein decreased, while the levels of p21 and p53 protein increased in the PAME-treated hBM-MSCs. However, PAME treatment did not significantly affect apoptosis/necrosis, ROS generation, and the level of Cdc25C protein. PAME also induced intracellular acidosis and increased intracellular Ca^2+^ levels. Cotreatment with PAME and Na^+^/H^+^ exchanger inhibitors together further reduced the intracellular pH but did not affect the PAME-induced decreases of cell proliferation and increases of the cell population at the G_2_/M phase. Cotreatment with PAME and a calcium chelator together inhibited the PAME-increased intracellular Ca^2+^ levels but did not affect the PAME-induced cell proliferation inhibition and G_2_/M cell cycle arrest. Moreover, the half-life of p53 protein was prolonged in the PAME-treated hBM-MSCs. Taken together, these results suggest that PAME induced p53 stabilization, which in turn increased the levels of p53/p21 proteins and decreased the levels of Cdk1/cyclin B1 proteins, thereby preventing the activation of Cdk1, and eventually caused cell cycle arrest at the G_2_/M phase. The findings from the present study might help get insight into the physiological roles of PAME in regulating hBM-MSC proliferation.

## 1. Introduction

Mesenchymal stem cells (MSCs), found in bone marrow stroma, adipose, and many other tissues, are candidates for tissue regeneration due to their high proliferation rate and potential for multilineage differentiation [1]. Recent studies have suggested that MSCs may not only replace diseased tissues but also exert several trophic, regenerative, and anti-inflammatory effects [2]. However, the number of MSCs that can be obtained from a donor remains insufficient for cell therapy purpose [3]. Therefore, it is imperative to obtain the maximum number and expand the population in vitro in order to be practicable for use in clinical application.

Human bone marrow-derived MSCs (hBM-MSCs) have been studied extensively for many years and used in multiple clinical studies and trials. They are self-renewable and retain the potential to differentiate into pericytes, myofibroblasts, bone marrow stromal cells, osteocytes, osteoblasts, and endothelial cells, all of which support hematopoiesis and stable bone mass [4, 5]. In recent studies, gender and age show significant effect on the number of hBM-MSCs and their proliferative capacity [6, 7]. The decrease in the number of resident MSCs may be one of the most important factors responsible for reduction in bone formation and the subsequent increase in bone fragility [8].

Bone marrow-derived MSCs reside within specialized microenvironments. These stem cell niches are essential for preservation of their self-renewal and differentiation capacity [9, 10]. Bone marrow is composed of multiple cell types including adipocytes, which are one of the most abundant cell types in adult bone marrow and constitute approximately 15% of the bone marrow volume in young adults, rising up to 60% by the age of 65 years old [11]. It has been reported that the number of adipocytes correlates inversely with the hematopoietic activity of the bone marrow. Adipocyte-rich bone marrow has a decreased number of hematopoietic stem cells compared to the adipocyte-poor bone marrow [12]. These findings implicate that adipocytes are predominantly negative regulators in the bone marrow microenvironment.

It has been shown that the adipose tissue produces and secretes various adipokines and free fatty acids (FFA), which could potentially influence the bone marrow niche for tissue homeostasis and repair [13]. A recent study showed that perivascular adipose tissue can release palmitic acid methyl ester (PAME), causing vasorelaxation [14]. PAME is an endogenous fatty acid methyl ester (FAME), which has been reported to possess potent anti-inflammatory and antifibrotic activities [15–17]. However, the effects of PAME on hBM-MSC proliferation remain unclear.

p53 protein can induce both cell cycle arrest and cell death. The regulation of cell fate decision has been the focus of numerous studies. Cell cycle arrest driven by p53 requires the transcription of p21, which is a cyclin-dependent kinase inhibitor. The p53/p21 pathway has been shown to play a role in the modulation of the differentiation and proliferation of stem cells [18, 19]. In normal cells, p53 is kept at low levels by murine double minute 2 (Mdm2), which can bind to p53 and act as p53 ubiquitin ligase that negatively regulates p53 function in several cellular pathways, such as cell cycle and apoptosis. Previous studies have shown that the Mdm2 inhibitor can decrease cell proliferation in rat BM-MSCs (rBM-MSCs) and hBM-MSCs [20, 21]. However, the mechanism underlying PAME-induced p53 stabilization and consequent cell cycle arrest in hBM-MSCs has not been elucidated. In the present investigation, we delineated the signaling pathway involved in the PAME-inhibited hBM-MSC proliferation.

## 2. Materials and Methods

### 2.1. Chemicals

PAME and stearic acid methyl ester (SAME) were purchased from Sigma-Aldrich (St. Louis, MO, USA) and dissolved in 100% methanol. Antimycin A (AMA), cariporide (HOE 642), 1,2-bis(2-aminophenoxy)ethane-N,N,N′,N′-tetraacetic acid tetra (acetoxymethyl ester) (BAPTA-AM), cycloheximide (CHX), ionomycin, and cantharidin (CTD) were purchased from Sigma-Aldrich. SC79 was purchased from Cayman Chemical Company (Ann Arbor, MI, USA); LB-100 was purchased from BioVision (Milpitas, CA, USA); ethyl isopropyl amiloride (EIPA) was purchased from Research Biochemical Incorporated (Natick, MA, USA).

### 2.2. Preparation and Culture of rBM-MSCs

Adult male Sprague-Dawley rats (270-350 g) were used as bone marrow cell donors. The rBM-MSCs were obtained from the tibias and the femurs of rats that were anesthetized using urethane (1.5 g/kg, i.p.) and sacrificed. The protocol was approved by the Institution of Animal Care and Use Committee of the Tzu Chi University (IACUC Approval No. 107003). Bone marrow cells were flushed out using phosphate-buffered saline (PBS) containing 1% penicillin/streptomycin (Gibco; Grand Island, NY, USA) and passed through a 100 *μ*m nylon gauze. The mononuclear rBM-MSCs were isolated by Ficoll density gradient centrifugation on Ficoll-Paque™ PLUS (GE Healthcare; Uppsala, Sweden). The mononuclear rBM-MSCs were pipetted, gently washed with PBS, and then centrifuged twice for 5 min at 1,200 rpm. The final pellet was resuspended in *α*-MEM (Gibco) supplemented with 15% fetal bovine serum (FBS) (Gibco) and 1% penicillin/streptomycin. The rBM-MSCs were incubated at 37°C in a fully humidified atmosphere with 5% CO_2_, and the medium was changed every 72 h. After growing to 70-80% confluence, the cells were detached using 0.25% trypsin-EDTA (Gibco) and then neutralized by adding fresh medium. For experiments, rBM-MSCs were cultured in *α*-MEM supplemented with 1% FBS, 1% penicillin/streptomycin, and various concentrations (10, 30, 50, or 100 *μ*M) of PAME for 48 h and incubated at 37°C in a fully humidified atmosphere with 5% CO_2_.

### 2.3. hBM-MSC Culture

The hBM-MSCs obtained from Sigma-Aldrich were cultured in *α*-MEM supplemented with 15% FBS and 1% penicillin/streptomycin. For experiments, hBM-MSCs were cultured in *α*-MEM supplemented with 1% FBS, 1% penicillin/streptomycin, and various concentrations (10, 30, 50, or 100 *μ*M) of PAME for 48 h and incubated at 37°C in a fully humidified atmosphere with 5% CO_2_.

### 2.4. MTT Assay

3-(4,5-Dimethylthiazol-2-yl)-2,5-diphenyl tetrazolium bromide (MTT) (Sigma-Aldrich) solution (5 mg/mL in PBS) was added to each 3.5 cm petri dish and incubated for 2 h at 37°C. Then, 1 mL of dimethyl sulfoxide (DMSO) (Sigma-Aldrich) was added to dissolve formazan crystals. The absorbance of each well was measured at 570 nm using an ELISA plate reader (Multiskan EX; Thermo, Waltham, MA, USA).

### 2.5. Hoechst 33342 Staining

The cells were fixed in 10% formaldehyde for 1 h at room temperature (RT) and then permeabilized for 15 min at RT with 0.15% Triton X-100. After blocking with 1% bovine serum albumin (BSA) in PBS for 15 min at RT, nuclei were stained with 2 *μ*M Hoechst 33342 (Sigma-Aldrich) and then incubated for 15 min at RT. Images were taken at 40x magnifications using a confocal microscope (Nikon C2 Si^+^; Melville, NY, USA) and analyzed using NIS-Elements imaging software.

### 2.6. BrdU Assay

Cell proliferation was evaluated by measuring 5-bromo-2′-deoxyuridine (BrdU) incorporation using a cell proliferation assay kit (Millipore; Burlington, MA, USA) following the manufacturer's instructions. Briefly, BrdU was added to each well of the 96-well plate and incubated for 24 h. The cells were washed with PBS for several times and then fixed with fixing solution for 30 min at RT, washed intensively, and then incubated with anti-BrdU monoclonal antibody for 1 h at RT. After washing, the cells were incubated with peroxidase-conjugated goat anti-mouse IgG for 30 min at RT, washed with PBS, and then incubated in TMB peroxidase substrate for 30 min at RT. The plate was read at 450 nm by ELISA plate reader (Multiskan EX; Thermo).

### 2.7. Western Blot Analysis

Proteins were extracted from hBM-MSCs and lysed in RIPA lysis buffer (Millipore, USA) containing 1% protease inhibitor (Calbiochem; San Diego, CA, USA) and 0.5% phosphatase inhibitor (Calbiochem). Protein concentration was determined using a BSA Protein Assay kit (Bio-Rad; Hercules, CA, USA). Electrophoresis sample buffer (1 M Tris-HCl, pH 6.8, 5% 2-mercaptoethanol, 20% glycerol, 10% SDS, and 0.5% bromophenol blue) was added to the cell lysates and boiled for 10 min at 110°C. Then, 30 *μ*g of the protein sample was loaded into a 10% SDS-polyacrylamide gel, subjected to electrophoresis, and then transferred to PVDF membrane (Millipore). The membrane was treated with 5% BSA in tween-tris-buffered saline (T-TBS) buffer (0.05% Tween 20, 200 mM Tris-HCl, pH 7.4, and 1.5 M NaCl) for 1 h at RT to block the nonspecific IgGs and then incubated with primary antibodies diluted in T-TBS buffer overnight at 4°C. The information of primary and secondary antibodies used in this study is shown as follows: primary antibodies including anti-Cdk1, anti-p21, anti-cyclin B1, anti-Cdc25C, anti-p53, and anti-Mdm2 were in 1,000 dilution; anti-*β*-actin purchased from GeneTex (Irvine, CA, USA) was in 10,000 dilution. Secondary antibodies including peroxidase-conjugated goat anti-rabbit IgG and anti-mouse IgG were diluted in T-TBS buffer (1 : 5,000). The resulting bands were analyzed using Image-Pro Plus software and normalized to *β*-actin.

### 2.8. Cytosolic Reactive Oxygen Species Analysis

hBM-MSCs were incubated with 2.5 *μ*M CM-H_2_DCFDA (Molecular Probes; Eugene, OR, USA) for 30 min at 37°C. Images were objective at 20x magnifications using a confocal microscope (Nikon C2 Si^+^) and analyzed using NIS-Elements imaging software.

### 2.9. Quantitative Real-Time PCR

Total RNAs were extracted from hBM-MSCs using TRIzol reagent (Ambion; Carlsbad, CA, USA) according to the manufacturer's instructions. Total cDNAs were synthesized with Verso™ cDNA Kit (Thermo) using 3 *μ*g of total RNAs. Quantitative real-time PCR was performed by mixing the cDNA with the Maxima SYBR Green qPCR Master Mix (2X) and ROX Solution (Thermo) and then detected using the ABI PRISM 7300 Real-Time PCR System (Applied Biosystems; Waltham, MA, USA). The following PCR primers were used: Cdk1, forward: 5′-TCA GGA TTT TCA GAG CTT TGG GCA CTC-3′, reverse: 5′-GCC ATT TTG CCA GAA ATT CGT TTG G-3′; cyclin B1, forward: 5′-TGC CCC TGC AGA AGA AGA CCT GTG T-3′, reverse: 5′-TGT TTC CAG CTT CCC GAC CCA GT-3′; p53, forward: 5′-CCC CTC CTG GCC CCT GTC ATC TTC-3′, reverse: 5′-GCA GCG CCT CAC AAC CTC CGT CAT-3′; p21, forward: 5′-GAG GCC GGG ATG AGT TGG GAG GAG-3′, reverse: 5′-CAG CCG GCG TTT GGA GTG GTA GAA-3′; and GAPDH, forward: 5′-TGC ACC ACC AAC TGC TTA GC-3′, reverse: 5′-GGC ATG GAC TGT GGT CAT GAG-3′. All gene expression was analyzed using the comparative Ct method (2^-*ΔΔ*Ct^), where ΔΔCt = ΔCt (sample) − ΔCt (reference) relative to GAPDH levels.

### 2.10. Flow Cytometry

Cell death, ROS, and intracellular pH analyses were carried out using FACSCalibur Flow Cytometer (Becton Dickinson Biosciences; San Jose, CA, USA). Cell cycle and intracellular Ca^2+^ analyses were carried out using Gallios™ Flow Cytometer (Beckman Coulter; Brea, CA, USA).

#### 2.10.1. Cell Death Analysis

Apoptosis/necrosis was examined using Annexin V-FITC Apoptosis Kit Plus (BioVision). The excitation/emission was detected at 488/530 nm wavelength.

#### 2.10.2. Cell Cycle Analysis

hBM-MSCs were dissociated into single cells with 0.25% trypsin, fixed in 1 mL 70% ethanol for 1 h at -20°C, and then centrifuged for 5 min at 2,000 rpm. The supernatant was removed, and Triton X-100 (0.1%) was added to permeabilize the hBM-MSCs, which were then treated with DNase-free RNase A (0.2 mg/mL) (Sigma-Aldrich) and stained with propidium iodide (PI) (20 *μ*g/mL) for 30 min at RT. The excitation/emission was detected at 488/585 nm wavelength.

#### 2.10.3. Reactive Oxygen Species Analysis

hBM-MSCs were incubated with 2.5 *μ*M MitoSOX™ Red reagent (Molecular Probes). The excitation/emission was detected at 510/580 nm wavelength.

#### 2.10.4. Intracellular Ca^2+^ Analysis

hBM-MSCs were incubated with 2.5 *μ*M Fluo-3-AM (Invitrogen; Carlsbad, CA, USA). The excitation/emission was detected at 488/525 nm wavelength.

#### 2.10.5. Intracellular pH Analysis

hBM-MSCs were incubated with 200 nM 2′,7′-bis(carboxyethyl)-5,6-carboxyfluorescein acetoxymethyl ester (BCECF-AM; Molecular Probes). The calibration buffer (140 mM KCl, 2 mM CaCl_2_, 1.2 mM MgSO_4_, 10 mM HEPES, 11 mM glucose, and 10 *μ*M nigericin) was adjusted to pH 6.5 with KOH. Nigericin, a K^+^/H^+^ ionophore, was used to set the internal pH to the external pH in the absence of a K^+^ gradient across the cell membrane so that [K^+^]_i_/[K^+^]_o_ equals to [H^+^]_i_/[H^+^]_o_. The excitation/emission was detected at 488/530 nm wavelength.

### 2.11. GC-MS Analysis

The rat bone marrow flush solutions were flushed out using 0.025 mL/g body weight PBS supplemented with 1% penicillin/streptomycin and 0.025 mL/g body weight *α*-MEM supplemented with 1% FBS and 1% penicillin/streptomycin. The samples were extracted with methanol to solubilize the organic compounds, vortexed, sonicated, and collected by centrifuge according to a previous report [14]. The supernatant was transferred to screw cap tubes with polytetrafluoroethylene/silicone septa in the caps. Samples were analyzed using a Hewlett-Packard (HP, Palo Alto, CA, USA) 6890 series II chromatograph coupled to a HP 5973 mass detector equipped with a G1512A automatic injector with BPX5 5% phenyl polysilphenylene-siloxane capillary column (25 m × I.D.0.22 mm; film thickness 0.25 *μ*m). The ionization energy was 70 eV. The carrier gas was He (flow 0.6 mL/min). The temperature of the injection block was 250-300°C. The GC oven temperature was programmed as follows: initial temperature 90°C followed by a temperature increase of 15°C/min up to 240°C and second rate of 10°C/min to the final temperature of 300°C. The mass spectrum was obtained by scanning from *m*/*z* 50 to 550. Data were acquired and analyzed using Hewlett-Packard G1701AA version 0.300 ChemStation Software.

### 2.12. Statistical Analyses

Experimental data was presented as means ± SEM and compared with unpaired *t*-tests. Data obtained from three or more groups were subjected to one-way ANOVA followed by Fisher's least significant difference test, and *p* values < 0.05 were considered significant.

## 3. Results

### 3.1. Detection of PAME and SAME in Rat Bone Marrow

**Figure 1:**
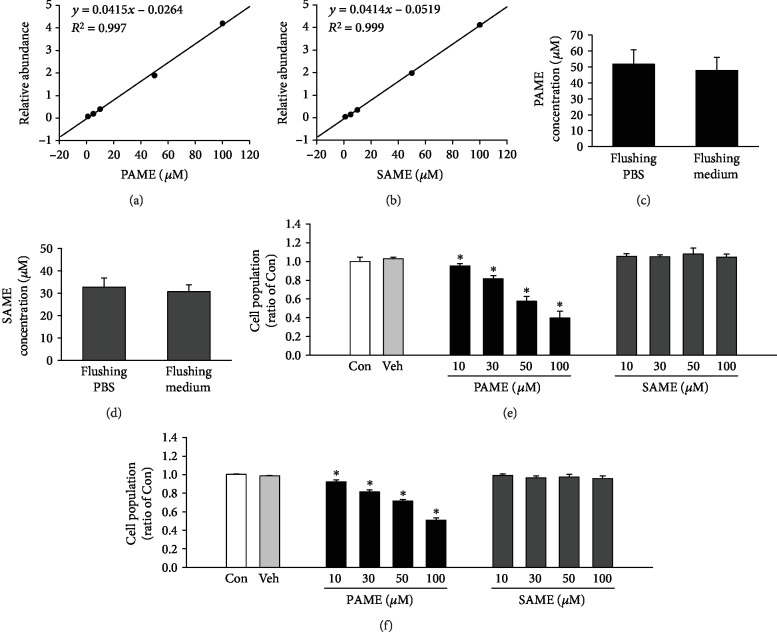
Detection of FAMEs in rBM and effects of FAMEs on cell proliferation in rBM-MSCs and hBM-MSCs. Calibration curves were generated for quantification of PAME (a) and SAME (b) using methanol solutions containing PAME and SAME in five different concentrations (1 *μ*M, 5 *μ*M, 10 *μ*M, 50 *μ*M, and 100 *μ*M). Both flushing PBS and flushing medium contain PAME (c) and SAME (d) from rBM (*n* = 3). PAME (10-100 *μ*M), but not SAME, concentration-dependently inhibited proliferation of rBM-MSCs (*n* = 3-9) (e) and hBM-MSCs (*n* = 5-15) (f). All data represent mean ± SEM. ^∗^*p* < 0.05, versus the control group. Con: control; rBM: rat bone marrow; Veh: vehicle.

Previous studies indicated that FAMEs including PAME and SAME were released from the superior cervical ganglion and retina [22, 23]. Initially, we used GC-MS to generate a five-point calibration curve obtained from analysis of methanol-extracted solutions containing a standard PAME and SAME in five different concentrations (1 *μ*M, 5 *μ*M, 10 *μ*M, 50 *μ*M, and 100 *μ*M), respectively (Figures 1(a) and 1(b)). This calibration curve was used for the quantitative analysis of PAME and SAME concentrations in the bone marrow flushing fluid. The rat femurs and tibias were excised, and the rat bone marrow (rBM) was flushed out with PBS. PAME and SAME in the bone marrow flushing PBS were detected and quantified by GC-MS. The PAME and SAME concentrations in the flushing PBS are 51.59 *μ*M and 32.66 *μ*M, respectively (Figures 1(c) and 1(d)). Previous studies have shown that the release of PAME from superior cervical ganglion, retinal, and perivascular adipose tissue is calcium-dependent [14, 22, 23]. We also used the medium to harvest the rBM, to determine whether the concentration of PAME and SAME in the flushing medium is different from those flushed with PBS. Our results show that the PAME and SAME concentrations in the flushing medium are 44.44 *μ*M and 30.87 *μ*M, respectively (Figures 1(c) and 1(d)), and they are not statistically significantly different from the concentrations obtained from the flushing PBS. These data indicate that the bone marrow contains FAMEs, including PAME and SAME.

### 3.2. Effects of PAME on rBM-MSC Proliferation

The effect of PAME on rBM-MSC proliferation was evaluated using the MTT assay. As shown in Figure 1(e), treatment with PAME (10-100 *μ*M) in medium containing 1% FBS for 48 h significantly reduced rBM-MSC proliferation in a concentration-dependent manner. To further examine the specificity of PAME on rBM-MSC proliferation inhibition, the proliferation effect of SAME, a structural analog of FAME, was tested. Treatment with SAME at a range of concentrations (10-100 *μ*M) for 48 h did not significantly affect the proliferation of rBM-MSCs (Figure 1(e), right panel). Methanol (1 : 1,000), the vehicle used in the incubation medium, did not significantly affect the viability of rBM-MSCs (Figure 1(e), left panel).

### 3.3. Effects of PAME on hBM-MSC Proliferation, Apoptosis, and Necrosis

**Figure 2:**
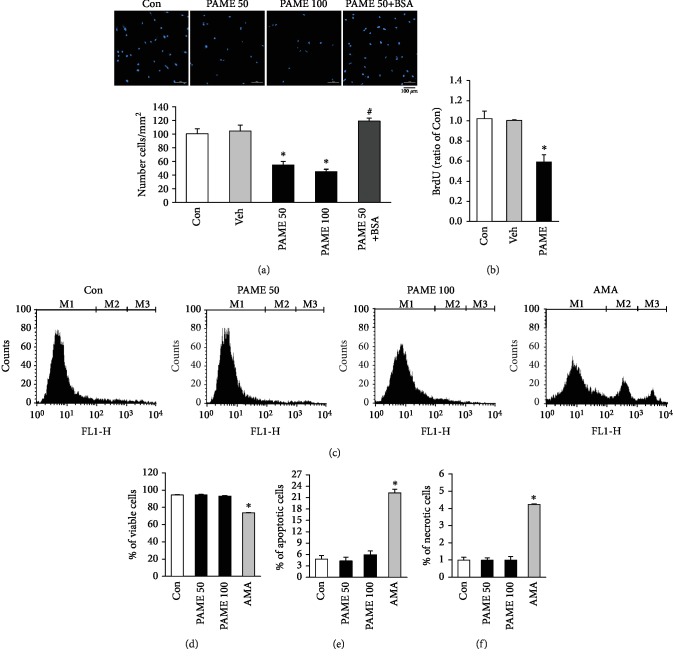
Effects of PAME on cell growth and death in hBM-MSCs. (a) Treatment with PAME (50 and 100 *μ*M) for 48 h inhibited hBM-MSC proliferation. This inhibition was blocked by 0.5% BSA (*n* = 3). (b) PAME (50 *μ*M)-induced proliferation inhibition in hBM-MSCs was confirmed by BrdU assay (*n* = 7). (c) Histogram plots of viable (M1), apoptotic (M2), and necrotic cells (M3) distinguished by flow cytometric analysis using Annexin V-FITC and SYTOX green dye staining. The (d) viable cells, (e) apoptotic cells, and (f) necrotic cells were not significantly different between the PAME-treated group and the control group (*n* = 6). AMA served as a positive control for cell death induction (*n* = 3). All data represent mean ± SEM. ^∗^*p* < 0.05, versus the control group; ^#^*p* < 0.05, versus the PAME group. Con: control; AMA: antimycin A; BSA: bovine serum albumin; Veh: vehicle.

The effect of PAME on the proliferation of hBM-MSCs was evaluated using MTT, Hoechst 33342 staining, and BrdU assay. In order to examine the proper FBS concentrations in the culture medium required for the PAME-induced proliferation inhibition, hBM-MSCs were incubated in the culture medium supplemented with PAME (50 *μ*M) and various concentrations of FBS (0%, 1%, 3%, 5%, 10%, and 15%) for 48 h. Our results show that PAME in the culture medium containing 1% or 3% FBS significantly inhibited hBM-MSC proliferation, and PAME had the strongest inhibition in the medium containing 1% FBS ([Supplementary-material supplementary-material-1]). Furthermore, treatment with PAME (10-100 *μ*M) in the medium containing 1% FBS for 48 h significantly reduced the proliferation of hBM-MSCs in a concentration-dependent manner (Figure 1(f)). On the other hand, treatment with SAME (10-100 *μ*M) in the medium containing 1% FBS for 48 h did not significantly affect hBM-MSC proliferation (Figure 1(f), right panel). Methanol (1 : 1,000), the vehicle used in the incubation medium, did not significantly affect the viability of hBM-MSCs (Figure 1(f), left panel). We also evaluated the effect of the PAME on the growth of hBM-MSCs by Hoechst 33342 staining assay. As shown in Figure 2(a), treatment with 50 or 100 *μ*M PAME for 48 h significantly reduced the number of hBM-MSCs. The inhibitory effect of PAME on the hBM-MSC proliferation was further confirmed by immunocytochemical detection of BrdU incorporation. As illustrated in Figure 2(b), BrdU incorporation, a DNA synthesis indicator, was significantly reduced in the hBM-MSCs exposed for 48 h to 50 *μ*M PAME, but not vehicle. To confirm that the PAME-induced reduction in the number of hBM-MSCs is not due to cell apoptosis and necrosis were examined by flow cytometry using Annexin V-FITC Apoptosis Kit Plus, which includes Annexin V-FITC for detecting apoptosis and SYTOX green dye for detecting necrosis.  Figure 2(c) shows the representative histograms of the PAME-treated hBM-MSCs stained with Annexin V-FITC Apoptosis Kit Plus. Treatment of hBM-MSCs with 50 or 100 *μ*M PAME for 48 h did not significantly affect the number of viable cells (Figure 2(d)), apoptotic cells (Figure 2(e)), and necrotic cells (Figure 2(f)). In contrast, treatment for 3 h with 20 *μ*M AMA, which served as a positive control for cell death induction, reduced the number of viable cells and increased the number of apoptotic and necrotic cells. These results indicate that the PAME-reduced hBM-MSC cell number was due to proliferation inhibition, but not induction of cell death.

### 3.4. Effects of PAME on hBM-MSC Cell Cycle Progression

**Figure 3:**
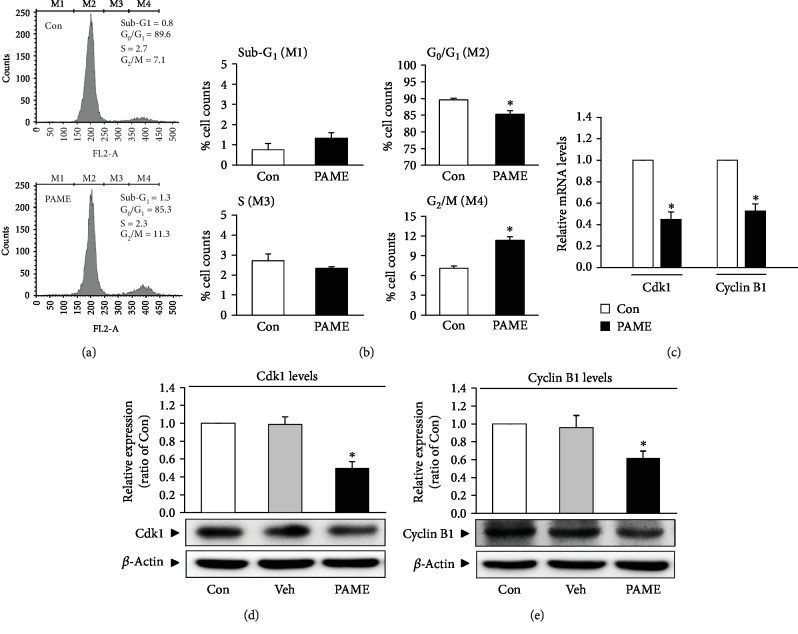
PAME induces G_2_/M cell cycle arrest in hBM-MSCs. The cells were treated with PAME (50 *μ*M) for 48 h prior to the flow cytometric and Western blot analyses. (a) Representative histograms of cell distribution at the sub-G_1_ phase (M1), G_0_/G_1_ phase (M2), S phase (M3), and G_2_/M phase (M4) detected by flow cytometry using PI staining. (b) PAME (50 *μ*M) significantly increased the cell population at the G_2_/M phase (M4) and decreased at the G_0_/G_1_ phase (M2) (*n* = 8). PAME significantly decreased the levels of (c) Cdk1 and cyclin B1 mRNA (*n* = 8) and (d, e) protein (*n* = 5). The levels of mRNA and protein were examined by qRT-PCR and Western blot analyses, respectively. All data represent mean ± SEM. ^∗^*p* < 0.05, versus the control group. Con: control; Veh: vehicle; PI: propidium iodide.

**Figure 4:**
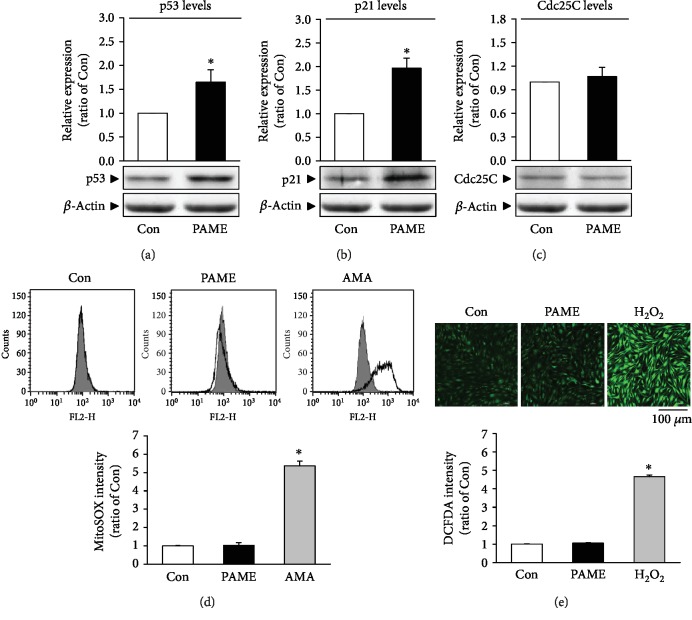
Involvement of the p53-p21 pathway and ROS in the PAME*-*induced G_2_/M cell cycle arrest in hBM-MSCs. Treatment with PAME (50 *μ*M) for 48 h increased the protein levels of (a) p53 (*n* = 9) and (b) p21 (*n* = 11), but not (c) Cdc25C (*n* = 8). (d) PAME did not significantly affect the level of mitochondrial ROS. The top panel shows that the mitochondrial ROS was detected by flow cytometric analysis using MitoSOX™ Red reagent; the bottom panel shows a graph of quantitation of these data. AMA-treated hBM-MSCs were used as a positive control for mitochondrial ROS production (*n* = 5). (e) PAME did not significantly affect the level of cytosolic ROS. H_2_O_2_-treated hBM-MSCs served as a positive control for cytosolic ROS production. The top panel shows ROS image detected by confocal microscopy using CM-H_2_DCFDA; the bottom panel shows a graph of quantitation of these data. At least 20 cells from 2 randomly selected fields were scored in each experiment to determine the DCFDA intensity (*n* = 5). All data represent mean ± SEM. ^∗^*p* < 0.05, versus the control group. AMA: antimycin A.

The effect of hBM-MSCs on cell cycle was investigated using PI for nucleic acid staining followed by flow cytometric analysis. PAME (50 *μ*M) significantly increased the cell population at the G_2_/M phase and decreased in the G_0_/G_1_ phase but did not significantly affect the S phase and the sub-G_1_ phase (Figures 3(a) and 3(b)). These results suggest that PAME induced cell cycle arrest at the G_2_/M phase. To confirm that PAME induced the G_2_/M phase arrest, the mRNA and protein levels of the G_2_/M regulatory proteins, Cdk1 and cyclin B1, were examined in hBM-MSCs. Quantitative RT-PCR assay demonstrated that the mRNA levels of both Cdk1 and cyclin B1 were significantly decreased in the PAME-treated group as compared with the control group (Figure 3(c)). The protein levels of Cdk1 (Figure 3(d)) and cyclin B1 (Figure 3(e)) detected by Western blot analyses also decreased in the PAME-treated group. Methanol (1 : 1,000), the vehicle used in the incubation medium, did not significantly affect the protein levels of Cdk1 (Figure 3(d)) and cyclin B1 (Figure 3(e)). Moreover, treatment with PAME significantly increased the protein levels of p53 (Figure 4(a)) and p21 (Figure 4(b)), but not Cdc25C (Figure 4(c)) in hBM-MSCs.

### 3.5. Effects of PAME on ROS Production

Cellular generation of ROS is central to redox signaling. Since ROS have been demonstrated to act as upstream signals triggering the p53 activation, we next examined the mitochondrial ROS production in the PAME-treated hBM-MSCs by flow cytometric analysis using MitoSOX™ Red reagent. As shown in Figure 4(d), treatment with PAME (50 *μ*M) in 1% FBS-containing medium for 48 h did not significantly affect the mitochondrial ROS level in hBM-MSCs. We also examined the cytosolic ROS production in hBM-MSCs treated with PAME (50 *μ*M) by confocal microscopy using CM-H_2_DCFDA. As demonstrated in Figure 4(e), PAME did not significantly affect the cytosolic ROS level. In these studies, the productions of ROS in the hBM-MSCs treated with 100 nM AMA for 30 min and 100 *μ*M H_2_O_2_ for 1 h were used as a positive control for ROS production, respectively.

### 3.6. Effects of Intracellular Acidosis on the PAME-Inhibited hBM-MSC Cell Proliferation

**Figure 5:**
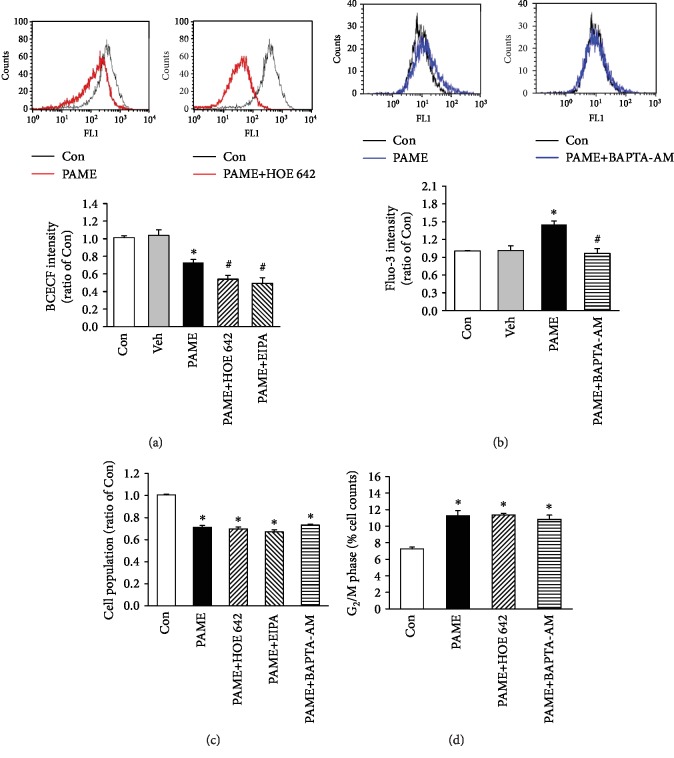
Involvement of intracellular acidosis and Ca^2+^ in the PAME-inhibited hBM-MSC proliferation. (a) Treatment with PAME (50 *μ*M) for 48 h induced the intracellular acidosis, and this effect was enhanced by cotreatment with a Na^+^/H^+^ exchanger blocker, HOE 642 or EIPA. The top panel shows the BCECF intensity, an indicator of cytosolic pH, detected using flow cytometric analysis; the bottom panel shows a graph of quantitation of these data (*n* = 6). (b) Treatment with PAME (50 *μ*M) for 48 h increased the intracellular Ca^2+^, and this effect was abolished by cotreatment with BAPTA-AM, a Ca^2+^ chelator. The top panel shows the Fluo-3 intensity, a fluorescence indicator of intracellular Ca^2+^; the bottom panel shows a graph of quantitation of these data (*n* = 6). (c) The PAME-decreased cell population was not significantly affected by cotreatment with HOC 642 or EIPA or BAPTA-AM. The cell population was detected by MTT assay (*n* = 8-20). (d) The PAME-increased cell accumulation at the G_2_/M phase was not significantly affected by cotreatment with HOE 642 or BAPTA-AM. The cell population at the G_2_/M phase was detected by flow cytometry using PI staining (*n* = 4). All data represent mean ± SEM. ^∗^*p* < 0.05, versus the control group; ^#^*p* < 0.05, versus the PAME group. Con: control; Veh: vehicle; EIPA: ethyl isopropyl amiloride; HOE 642: cariporide; PI: propidium iodide.

Since arachidonic acid methyl ester (AAME) has been reported to be able to induce intracellular acidosis in rat cardiac myocytes and cerebellar granule cells [24, 25], we examined whether PAME could induce intracellular acidosis, hence inhibiting hBM-MSC proliferation. Treatment of hBM-MSCs with PAME (50 *μ*M) for 48 h significantly reduced the level of BCECF, an intracellular ratiometric pH indicator. Moreover, cotreatment with PAME and a Na^+^/H^+^ exchanger blocker, cariporide (HOE 642) or ethyl isopropyl amiloride (EIPA), further reduced the level of BCECF (Figure 5(a)). Methanol (1 : 1,000), the vehicle used in the incubation medium, did not significantly affect the intracellular acidosis of hBM-MSCs (Figure 5(a)). However, cotreatment with PAME and HOE 642 or EIPA did not further reduce the PAME-induced reduction in the hBM-MSC cell number (Figure 5(c)). Moreover, cotreatment with PAME and HOE 642 together did not further increase the PAME-increased percentage of the cell number at the G_2_/M phase (Figure 5(d)). Taken together, these data suggest that the PAME-induced hBM-MSC proliferation inhibition was not due to intracellular acidosis.

### 3.7. Effects of Intracellular Ca^2+^ on the PAME-Inhibited hBM-MSC Cell Proliferation

Cytosolic Ca^2+^ concentrations fluctuate in an ordered manner along the cell cycle, suggesting that Ca^2+^ might be involved in regulating cell proliferation. Since p53 can modulate intracellular Ca^2+^ homeostasis [26], we examined whether PAME could affect the intracellular Ca^2+^ concentrations, hence inhibiting hBM-MSC proliferation. Treatment of hBM-MSCs with PAME (50 *μ*M) for 48 h significantly increased the level of Fluo-3, an intracellular Ca^2+^ indicator. Methanol (1 : 1,000), the vehicle used in the incubation medium, did not significantly affect the intracellular Ca^2+^ of hBM-MSCs (Figure 5(b)). Moreover, cotreatment with PAME and BAPTA-AM (0.5 *μ*M), a Ca^2+^ chelator, abolished the PAME-increased level of Fluo-3-AM (Figure 5(b)) but did not significantly affect the PAME-induced proliferation inhibition in hBM-MSCs (Figure 5(c)) and the PAME-increased percentage of the cell number at the G_2_/M phase (Figure 5(d)), suggesting that the PAME-induced hBM-MSC proliferation inhibition was not due to an increase of intracellular Ca^2+^ concentration.

### 3.8. Effects of PAME on p53 Protein Stabilization

**Figure 6:**
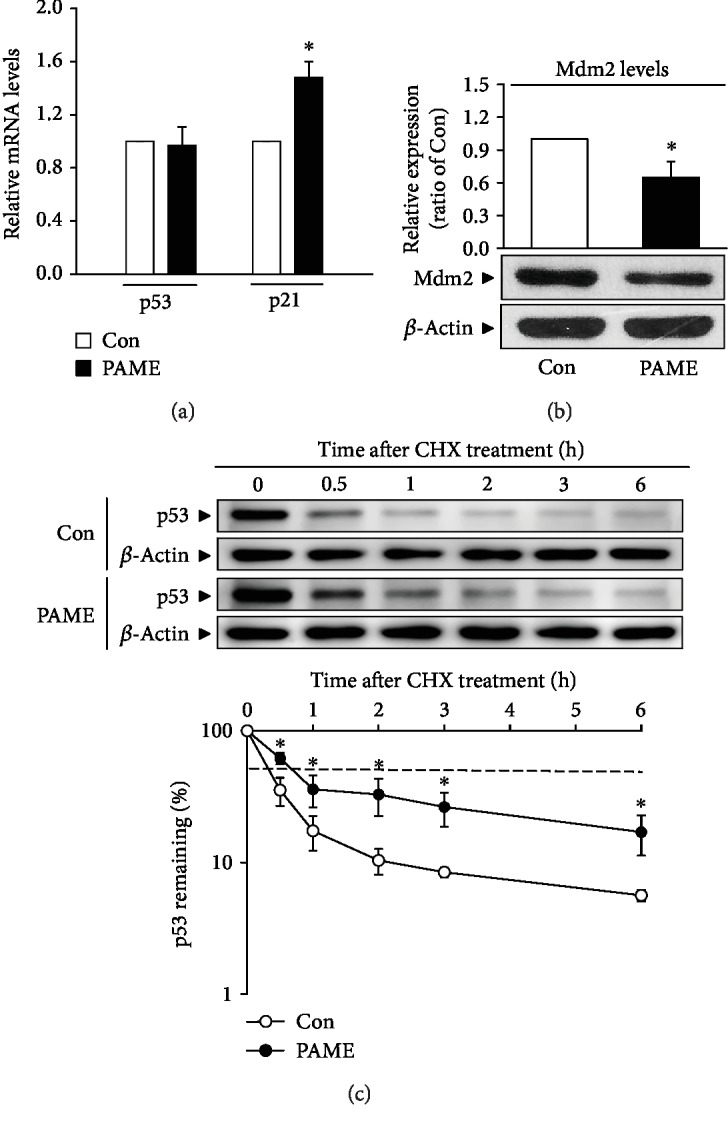
Effects of PAME on p53 protein stability. PAME significantly increased the (a) mRNA level of p21 but not p53 (*n* = 5) and reduced the (b) level of Mdm2 protein (*n* = 10). The mRNA levels were detected by qRT-PCR, and the protein level was determined by Western blot analysis. (c) PAME prolonged the degradation of p53 protein in hBM-MSCs. The dotted line indicates 50% abundance of p53, and the half-life (t1/2) of p53 protein in each group is shown in the bottom panel. hBM-MSCs were treated with PAME (50 *μ*M) for 6 h followed by CHX (5 *μ*M) treatment and then subjected to protein level detection by Western blot analysis (*n* = 4-6). All data represent mean ± SEM. ^∗^*p* < 0.05, versus the control group. Con: control; CHX: cycloheximide.

We further investigated whether PAME-increased levels of p53 and p21 proteins were due to increases of transcription and/or translation. The quantitative RT-PCR assay demonstrated that PAME significantly increased the mRNA level of p21, but not p53, in hBM-MSCs (Figure 6(a)). These findings suggest that PAME might increase the p53 protein stability. To address this issue, the levels of Mdm2 protein, a negative regulator of p53, and p53 protein degradation rate were examined. Western blot analyses showed that PAME (50 *μ*M) treatment reduced the protein levels of Mdm2 in hBM-MSCs (Figure 6(b)). Moreover, treatment with PAME for 6 h followed by cycloheximide (CHX), a protein synthesis inhibitor, for 0.5 to 6 h significantly prolonged the half-life of p53 protein (Figure 6(c)). Thus, PAME can indeed reduce the rate of p53 degradation, hence causing p53 protein stabilization.

## 4. Discussion

hBM-MSCs have been widely investigated for their potential therapeutic applications. However, the number of hBM-MSCs in bone marrow is not high enough for therapeutic purpose. Therefore, experimental and clinical investigators continue to search for new methods to expand the isolated hBM-MSCs in vitro for obtaining a sufficient number. In the present study, we demonstrated that PAME significantly reduced the proliferation in rBM-MSCs and hBM-MSC (Figures 1(e) and 1(f)). These findings are different from the results of two other groups [27, 28]. The discrepancy between our results and their results might be due to the difference in the PAME concentrations used. In our study, the concentration of PAME used is 50 *μ*M, which is close to the concentration detected in the flushing medium of rat bone marrow and did not cause cell death but significantly reduced the proliferation in both rBM-MSCs and hBM-MSCs (Figures 1 and 2). On the other hand, the concentration of PAME used in their study was 333 *μ*M or above, which is much higher than the concentration used in our study.

PAME, one of the most abundant fatty acids in mammalian cells [29], represents an endogenous naturally occurring FAME [30]. It has been reported that endogenous PAME is able to inhibit phagocytosis in primary rat Kupffer cells [15, 31]; to inhibit fibrotic effects [17, 32, 33], inflammation [16, 34], and oxidative stress [35]; to induce vasodilation [14, 22, 23, 36, 37]; and to prevent nonalcoholic steatohepatitis [38]. However, the molecular mechanisms underlying PAME-induced biologic effects are still unclear. In the present study, we demonstrated that treatment with PAME induced cell cycle arrest at the G_2_/M phase through upregulation of p53 protein, which has been indicated in regulating the stress-induced G_2_/M transition of the mitotic cell cycle. The mechanism by which p53 regulates the G_2_/M transition involves the regulation of Cdk1, which is essential for the cell cycle entry into mitosis [39]. Repression of cyclin B1 and Cdk1 by p53 enforces the arrest. However, p53-induced repression of cyclin B1 and Cdk1 might involve p21 upregulation. p21, a transcriptional target of p53, can inhibit the activity of Cdks and participate in the G_2_ checkpoint. p21 inhibits the Cdk activity by direct binding to the Cdk/cyclin complexes. Although p53 has other targets that do not affect the Cdk1 activity but also contribute to the G_2_ arrest, our data suggest that PAME induced G_2_/M cell cycle arrest through the p53/p21-mediated reduction of the protein levels of Cdk1 and cyclin B1 in hBM-MSCs (Figures 3, 4(a), and 4(b)). Previous studies have shown that PAME can enhance cerebral blood flow (CBF), thereby alleviating neuronal cell death in the CA1 region of the hippocampus after cerebral ischemia. Furthermore, PAME also exerts other nonvasodilation-dependent neuroprotective effects (i.e., antiapoptosis) [40, 41]. In the present study, our data revealed that PAME did not cause apoptosis and necrosis in hBM-MSCs (Figures 2(e) and 2(f)) but did increase the expression of p21 (Figure 4(b)), which has been indicated to act as an inhibitor of apoptosis in a number of systems [42]. The antiapoptosis/necrosis caused by accumulation of p21 might contribute to the PAME-induced neuronal protection after ischemia.

Previous studies have shown that PAME reduces CCl_4_-induced or lipopolysaccharide- (LPS-) induced phosphorylation of inhibitory kappa B (I*κ*B*α*), nuclear translocation of nuclear factor-*κ*B (NF-*κ*B), and subsequent proinflammatory cytokine production in vitro and in vivo [15, 16, 33, 34]. However, the mechanism underlying the PAME-regulated NF-*κ*B level has not been clarified. It has been shown that ectopic expression of p53 enhanced NF-*κ*B DNA binding but blocked its transactivation function [43, 44]. In the present study, we showed that PAME significantly increased the p53 protein level (Figure 4(a)), which may be involved in the PAME-reduced nuclear NF-*κ*B level and inflammatory response. On the other hand, NF-*κ*B can reduce the p53 levels by upregulating the Mdm2 expression [44, 45]. Therefore, PAME may upregulate the p53 protein level through downregulating the Mdm2 expression caused by reducing the nuclear translocation of NF-*κ*B.

Acidosis has been demonstrated to increase the p53 protein levels and the mRNA levels of p21 and Mdm2 [46]. In the present study, we demonstrated that PAME significantly induced intracellular acidosis in hBM-MSCs (Figure 5(a)). Maintenance of acid-base homeostasis in extracellular fluids and cytoplasm is essential for cell proliferation and differentiation. It has been shown that acidity reduces the proliferation and viability of rBM-MSCs and human adipose-derived MSCs [47, 48]. hBM-MSCs are highly sensitive to small shifts in external pH. It has been reported that incubation of PMC-22 cells at pH 6.5 medium for 48 h results in marked decreases of cell population at the S phase and accumulation of cells at the G_1_ phase [49]. Application of sublethal acidic stress (pH 3.3, 37°C) for 25 min induces G_1_ arrest in Jurkat T-lymphocytes and G_2_ arrest in A301 cells [50]. However, cotreatment with PAME and HOE 642 or EIPA together did not affect the PAME-induced G_2_/M arrest in hBM-MSCs (Figure 5(d)), suggesting that the PAME-induced G_2_/M arrest is not caused by intracellular acidosis induction.

Although PI staining can be used to assess cell cycle states, it cannot determine that the cells in the S phase are actually cycling. In Figure 2(b), we used BrdU to detect the progress of S phase; the results showed that PAME can significantly inhibit the DNA synthesis. Although using BrdU pulse/chase experiment can more easily identify cells in the G_1_, S, and G_2_ phases and define the cell cycle kinetics [51], this technique might not be suitable for examining the cell cycle kinetics in stem cells due to a very low ratio of stem cells in the S phase. We did try to detect the cell cycle kinetics of hBM-MSCs using the FITC BrdU Flow Kit (BrdU and 7-AAD), but the results showed a very low proportion of BrdU-positive cells. Therefore, prepulse with BrdU cannot chase the cell cycle kinetics effectively.

Intracellular Ca^2+^ rise is involved in controlling numerous cellular processes, such as cell proliferation, differentiation, and apoptosis. It has been reported that an increase of intracellular Ca^2+^ results in activation of p53/p21 and induces cell cycle arrest in melanoma cells [52]. Moreover, growth inhibition of human keratinocytes caused by high Ca^2+^ has been shown to be mediated by p21 upregulation [53, 54]. In the present study, we showed that PAME increased intracellular Ca^2+^ level (Figure 5(b)). However, cotreatment with PAME and BAPTA-AM, a Ca^2+^ chelator, did not affect the PAME-induced G_2_/M arrest in hBM-MSCs (Figure 5(d)), suggesting that the PAME-induced G_2_/M arrest is not caused by increases of the intracellular Ca^2+^. In addition, it has been indicated that p53 can directly bind to the sarco/endoplasmic reticulum Ca^2+^-ATPase (SERCA) pump at the sarco/endoplasmic reticulum (SR/ER), changing its oxidative state and thus leading to an increased Ca^2+^ load, followed by an enhanced transfer to mitochondria [55]. This signaling has also been recently proved to be important for M phase progression [56]. Unfortunately, the SR/ER to mitochondrial Ca^2+^ transmission is poorly sensitive to BAPTA-AM. In order to further rule out the changes of Ca^2+^ in SR/ER and mitochondria, which might affect M phase progression, we used Mag-Fluo4 and Rhod-2 to measure the SR/ER and mitochondrial Ca^2+^ concentration, respectively. The data show that PAME caused a significant increase in the SR/ER Ca^2+^ but did not significantly affect the mitochondrial Ca^2+^ ([Supplementary-material supplementary-material-1]). These data suggest that the PAME-induced G_2_/M arrest is not caused by increases of mitochondrial Ca^2+^. The physiological significance of PAME-increased intracellular acidosis and intracellular Ca^2+^ deserves further investigation.

Mdm2 has been demonstrated to play a major role in regulating hematopoietic stem cell survival [57]. Moreover, previous studies have shown that Mdm2 inhibitors can inhibit cell proliferation in rBM-MSCs and hBM-MSCs [20, 21]. Dysfunction of the Mdm2/p53 axis has been linked to cell proliferation and apoptosis. Cdc25C is a dual-specificity phosphatase that promotes cell cycle entry into mitosis by removing the inhibitory phosphates on Cdks. Mdm2-promoted Cdc25C protein degradation has been shown to delay cell cycle progression through the G_2_/M phase [58]. However, treatment with PAME did not significantly affect the levels of Cdc25C protein in hBM-MSCs (Figure 4(c)). In the present study, we demonstrated that treatment with PAME induced hBM-MSC cell cycle arrest at the G_2_/M phase via a p53-dependent pathway.

It has been shown that Akt, a protein serine/threonine kinase involved in regulating cell growth, proliferation, and apoptosis processes, can enhance the Mdm2-mediated ubiquitination and degradation of p53 [59]. Akt activation is controlled by protein phosphatase 2A (PP2A), a serine/threonine phosphatase involved in mitotic progression and cellular responses to DNA damage. A recent study demonstrated that PP2A leads to cell cycle arrest through the p53/p21 pathway in hepatocytes [60]. Another study provide evidence showing that PP2A blocks HA22T cell proliferation through suppressing the PI3K/Akt/Mdm2-mediated p53 activation [61]. In the present study, we showed that treatment with PAME significantly increased the protein level of PP2A and decreased the protein level of p-Akt. However, cotreatment with PAME and a PP2A inhibitor, cantharidin (CTD) or LB-100, or an Akt activator, SC79, did not affect the PAME-induced proliferation inhibition in hBM-MSCs ([Supplementary-material supplementary-material-1]). These findings suggest that activation of Akt and upregulation of PP2A might not participate in the PAME-induced hBM-MSC proliferation inhibition.

Although excessive proliferation of hBM-MSCs has not been reported to be associated with clinical disease, previous studies have shown that hBM-MSCs can be recruited to the tumor microenvironment, subsequently promoting proliferation, invasion, survival, tumorigenicity, and migration in a variety of cancers [62, 63]. Moreover, BM-MSCs enhance the chemoresistance of ovarian cancer by releasing miR-1180 and upregulating the glycolytic level [64]. Our data showed that PAME induced cell cycle arrest and reduced cell numbers in BM-MSCs (Figures 1(e), 1(f), 2(a), 2(b), and 3). We also found that PAME can increase the p53 expression in A549 cancer cell line ([Supplementary-material supplementary-material-1]). Therefore, use of PAME to reduce hBM-MSCs might be a potential application for treating cancer diseases.

## 5. Conclusions

**Figure 7:**
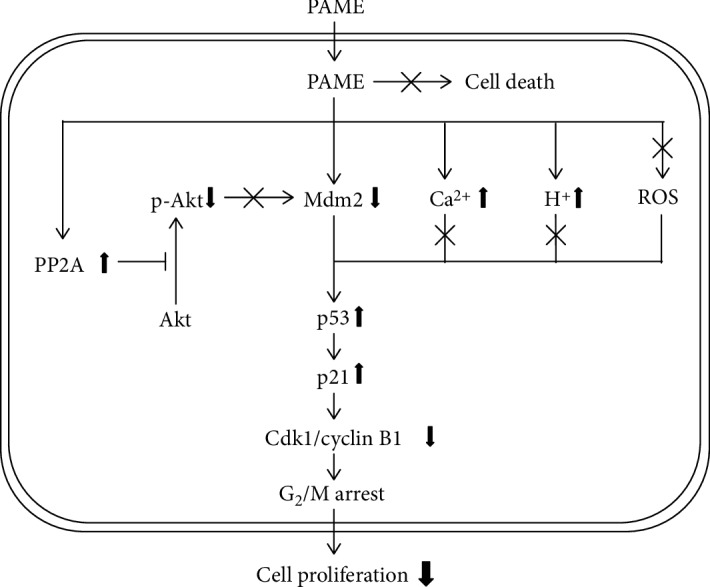
Schematic representation of the PAME-induced G_2_/M arrest in hBM-MSCs. PAME decreased the expression of Mdm2, which in turn reduced the degradation of p53, subsequently upregulating the expression of Cdk1 and cyclin B1 through suppression of the p21 protein level, and eventually caused cell cycle arrest at the G_2_/M phase.

Our results suggest that PAME downregulated Mdm2, which in turn caused p53 stabilization, subsequently increasing the protein levels of p53 as well as p21 and decreasing the levels of Cdk1/cyclin B1 protein, and eventually caused cell cycle arrest at the G_2_/M phase. We propose a model of the molecular mechanism underlying PAME-induced cell cycle arrest in hBM-MSCs as shown in Figure 7.

## Data Availability

All materials are available from the corresponding author.

## Supplementary Materials

Supplementary Materials Supplement 1: effects of FBS concentrations on the PAME-inhibited hBM-MSC proliferation. Supplement 2: effects of PAME on SR/ER and mitochondrial [Ca^2+^] in hBM-MSCs. Supplement 3: involvement of the Akt and PP2A in the PAME-inhibited hBM-MSC proliferation. Supplement 4: effects of PAME on the p53 protein level in A549 cells.
