# Supplementary material for: Palmitic Acid Methyl Ester Induces G2/M Arrest in Human Bone Marrow-Derived Mesenchymal Stem Cells via the p53/p21 Pathway
Source: Stem Cells Int. 2019 Dec 1;2019:7606238. doi: 10.1155/2019/7606238 (PMC6915012; doi:10.1155/2019/7606238)
Supplement: Supplementary Materials — Supplement 1: effects of FBS concentrations on the PAME-inhibited hBM-MSC proliferation. Supplement 2: effects of PAME on SR/ER and mitochondrial [Ca2+] in hBM-MSCs. Supplement 3: involvement of the Akt and PP2A in the PAME-inhibited hBM-MSC proliferation. Supplement 4: effects of PAME on the p53 protein level in A549 cells. [file 7606238.f1.docx]

**Supplement 1.**


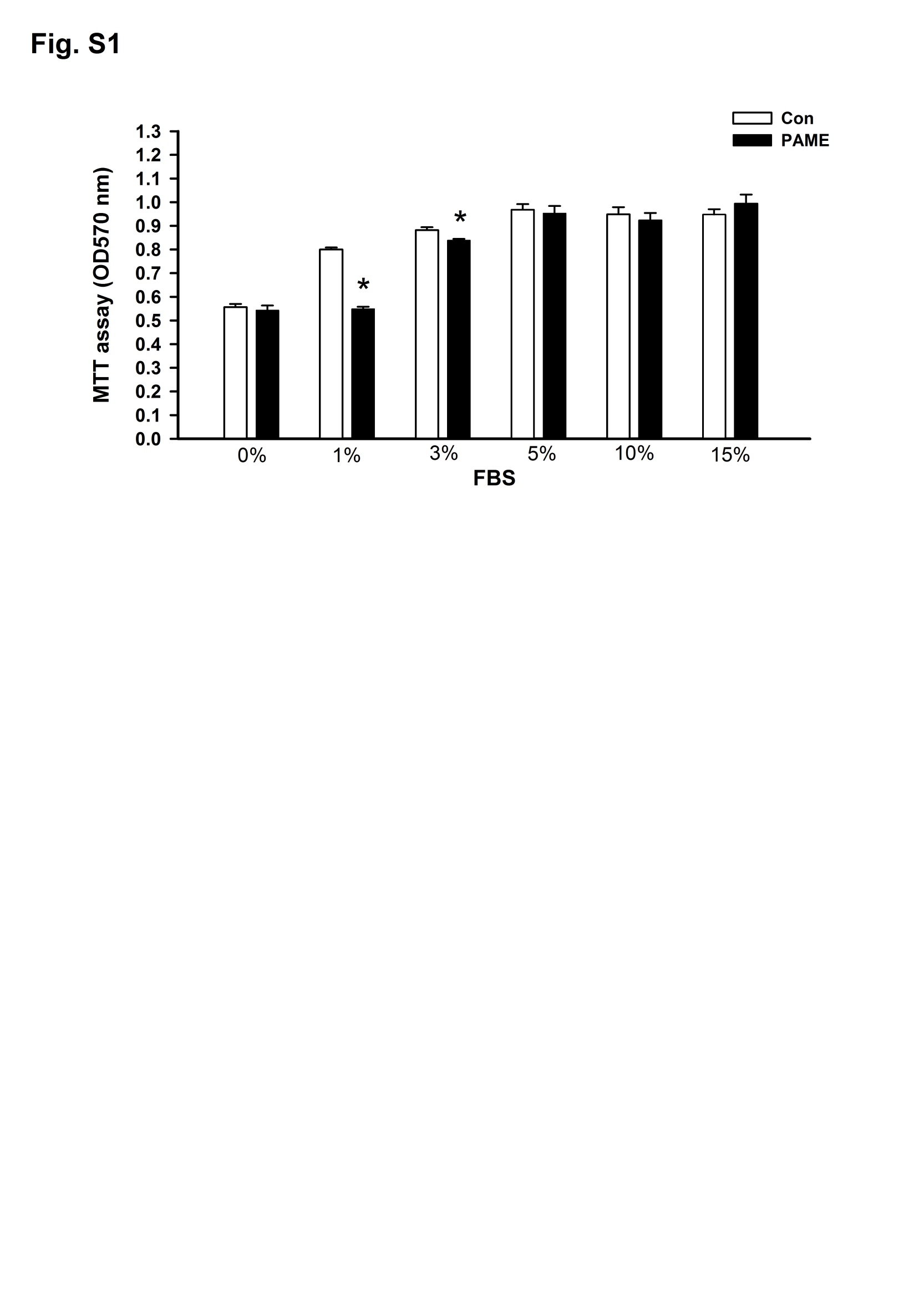


**Supp. 1:** Effects of FBS concentrations on the PAME-inhibited hBM-MSC proliferation. The hBM-MSC proliferation was evaluated by MTT assay. PAME (50 µM) significantly inhibited hBM-MSC proliferation in a medium containing 1% or 3% FBS (n = 3-6). All data represent mean ± SEM. **p* < 0.05, versus the control group. Con, control.

**Supplement 2.**

**
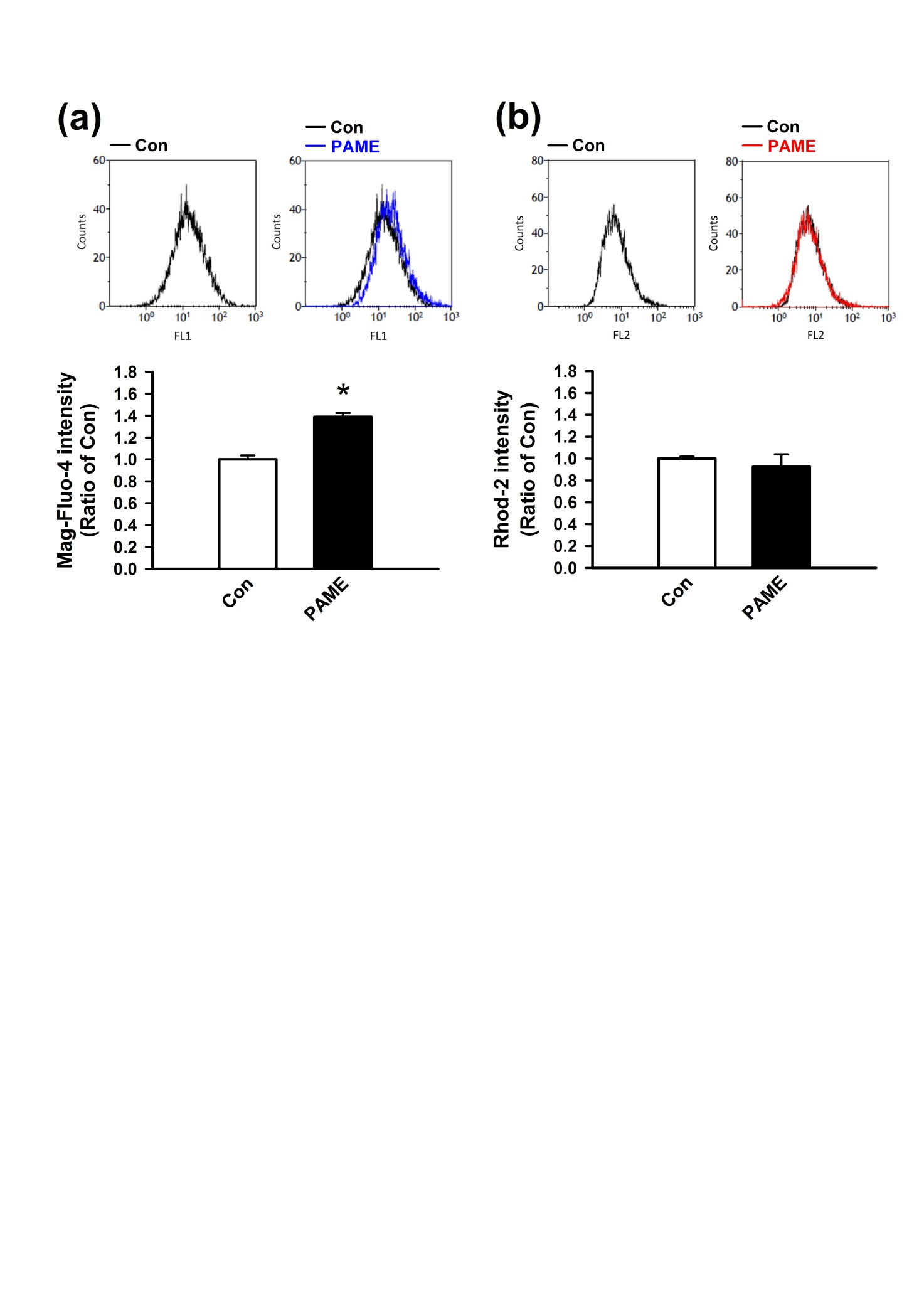
**

**Supp. 2:** Effects of PAME on SR/ER and mitochondrial [Ca^2+^] in hBM-MSCs. Treatment with PAME (50 μM) for 48 h increased the SR/ER [Ca^2+^], but not mitochondrial [Ca^2+^]. (a) Cells were preloaded with Mag-Fluo-4 (1 μM), an indicator of SR/ER [Ca^2+^], and then detected by flow cytometric analysis (Ex/Em = 494/527 nm). The top panel shows the representative results of Mag-Fluo-4 intensity; bottom panel shows the quantitative results of Mag-Fluo-4 intensity (n = 6). (b) Cells were preloaded with Rhod-2 (2.5 μM), an indicator of mitochondrial [Ca^2+^], and detected by flow cytometric analysis (Ex/Em = 552/581 nm). The top panel shows the representative results of Rhod-2 intensity; bottom panel shows the quantitative results of Rhod-2 intensity (n = 10). All data represent mean ± SEM. **p* < 0.05, versus the control group. Con, control.

**Supplement 3.**

**
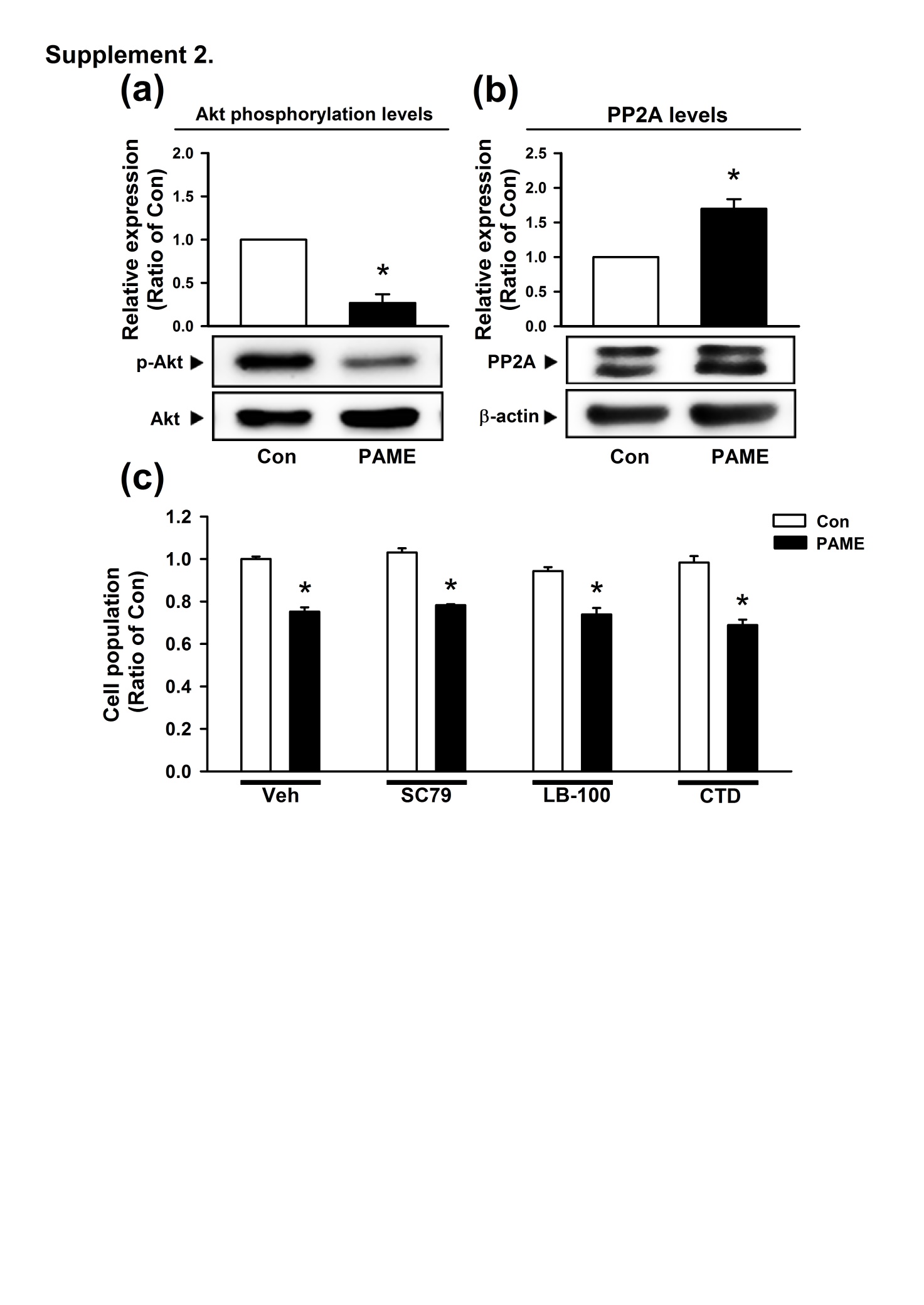
**

**Supp. 3:** Involvement of the Akt and PP2A in the PAME-inhibited hBM-MSC proliferation. The effects of PAME (50 μM) treatment on the levels of p-Akt and PP2A protein were evaluated by Western blot analysis. (a-b) PAME (50 μM) treatment increased the protein levels of p-Akt and PP2A. (n = 4). (c) The PAME-induced hBM-MSC proliferation inhibition were not significantly affected by SC79 (1 μM), LB-100 (50 nM) or CTD (100 nM) (n = 5-16). All data represent mean ± SEM. **p* < 0.05, versus the control group. Con, control; CTD, cantharidin.

**Supplement 4.**

**
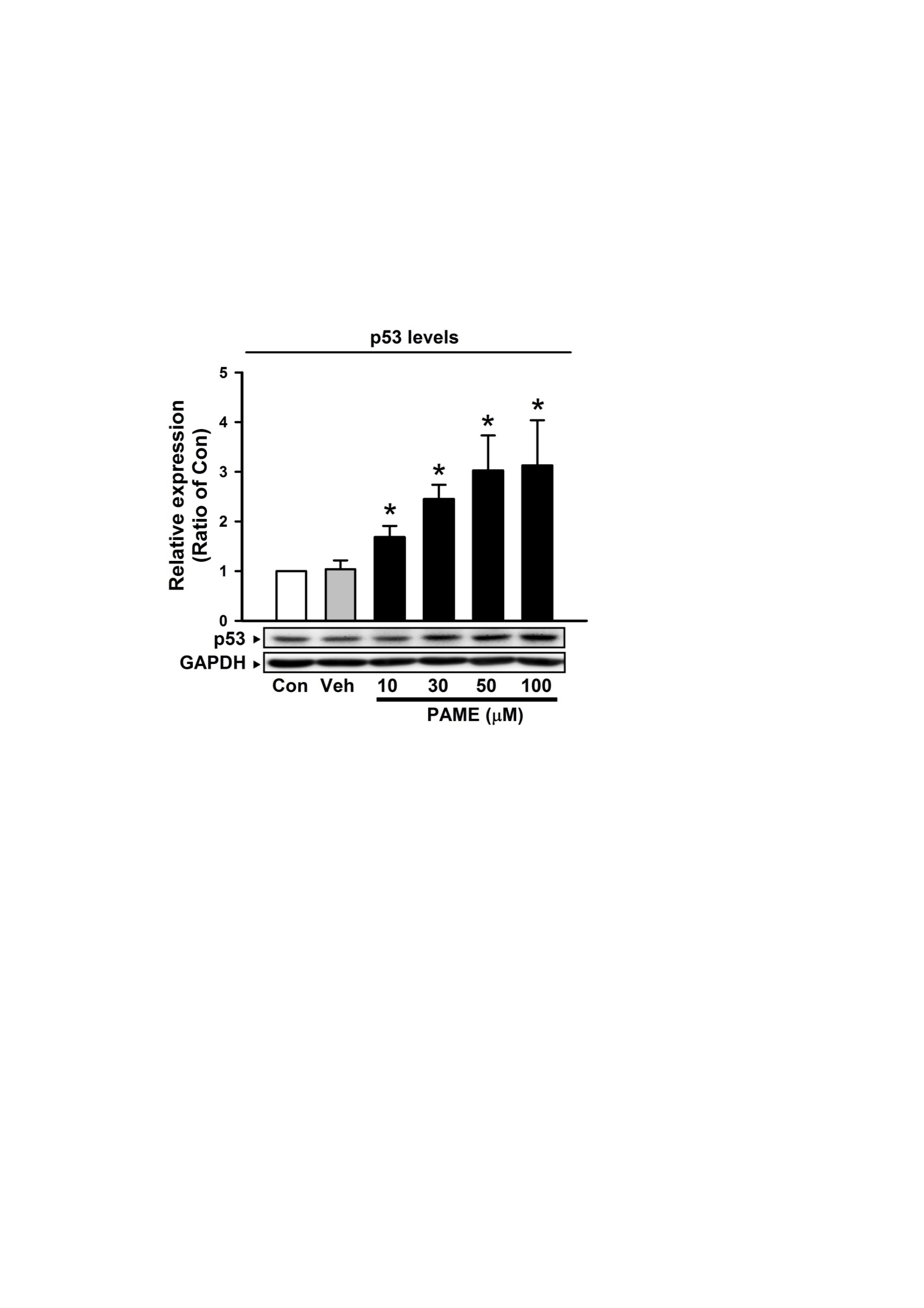
**

**Supp. 4:** Effects of PAME on the p53 protein level in A549 cells. A549 cells were treated with various concentrations of PAME (10-100 μM) in DMEM-high glucose supplemented with 1% FBS and 1% penicillin/streptomycin for 48 h. The protein level of p53 was measured by Western blot analysis. PAME (10-100 μM) concentration-dependently increased the p53 protein levels. (n = 6). All data represent mean ± SEM. **p* < 0.05, versus the control group. Con, control; Veh, vehicle.
